# Vaccination against SARS-CoV-2 and disease enhancement – knowns and unknowns

**DOI:** 10.1080/14760584.2020.1800463

**Published:** 2020-08-24

**Authors:** Raphaël M. Zellweger, T. Anh Wartel, Florian Marks, Manki Song, Jerome H. Kim

**Affiliations:** a Epidemiology, Public Health, Impact & Clinical Development Unit, International Vaccine Institute, Seoul, Republic of Korea; b Science Unit, International Vaccine Institute, Seoul, Republic of Korea

**Keywords:** Coronavirus, SARS-CoV-2, COVID-19, disease enhancement, vaccine safety

## Abstract

**Introduction:**

The world is currently fighting a COVID-19 pandemic, perhaps the most disruptive infectious disease outbreak since the 1918 Spanish influenza. Governments have taken drastic measures to curb the spread of SARS-CoV-2, and the development of safe and efficacious vaccine candidates is being accelerated. The possibility of vaccine-mediated disease enhancement with coronavirus vaccines has been flagged as a potential safety concern, and, despite the urgent need, should be thoroughly assessed as vaccines against SARS-CoV-2 are being tested.

**Area covered:**

We review the in vivo evidence suggesting a theoretical risk of disease enhancement after vaccination with SARS-CoV and MERS-CoV vaccine candidates. We also identify knowledge gaps that need to be filled to maximize the chance of developing a safe vaccine and minimize the risk of encountering disease enhancement in vaccinated individuals after exposure to SARS-CoV-2.

**Expert opinion:**

We compile and propose avenues to investigate the risk of vaccine-mediated disease enhancement both during pre-clinical and early clinical development. While the pressing need for a vaccine against COVID-19 (and future epidemic coronaviruses) cannot be ignored, we advocate to keep safety at the center of the debate. Protecting individuals with effective and safe vaccines should be a priority, even during extraordinary times like the COVID-19 pandemic.

## 1. Introduction

### 1.1. The need for a COVID-19 vaccine

In February 2018, the World Health Organization (WHO) enriched its Blueprint list of priority diseases with ‘Disease X’, an as-yet-unknown pathogen that could potentially cause a serious international epidemic [1]. Less than two years later, Disease X materialized in the form of SARS-CoV-2, a novel coronavirus (CoV) causing the current COVID-19 pandemic. Five months after the first cases in China, there were 6,722,408 confirmed cases, including 393,933 deaths, in 188 countries (as of 6 June 2020) [2]. The spread of COVID-19 has already impacted the world in an extraordinary way, prompting unprecedented control measures that profoundly disrupt daily lives for millions (if not billions) of individuals. Measures to mitigate the spread of the virus come at a high economic and societal cost. At present, countries around the world are still partly paralyzed by social restrictions and lockdowns, a dramatic reduction in air travel, a plummeting global economy, and health systems staggering under the burden of hospitalization and death. Therefore, the world is rushing for a vaccine to curb this pandemic. However, developing a vaccine against a novel and highly transmissible pathogen (for which knowledge is still emerging) in an accelerated framework poses unique challenges.

As of 5 June 2020, there were 167 vaccine candidates in pre-clinical development, 13 in phase I or II and 1 in phase II/III, including new platforms such as RNA and DNA vaccines (which are particularly attractive in outbreak situations [3]) as well as protein-subunit, inactivated, non-replicating and replicating viral vectors, and live-attenuated candidates [4,5]. While this is extremely encouraging, the possibility of vaccine-induced disease enhancement after vaccination against SARS-CoV-2 has been flagged as a potential safety concern that requires particular attention by the scientific community, including the WHO [6], the Coalition for Epidemic Preparedness Innovations (CEPI) [7] and the International Coalition of Medicines Regulatory Authorities (ICMRA) [8].

### 1.2. Vaccine-induced disease enhancement

Vaccine-induced disease enhancement is not a new concern for vaccine developers [9]. In 1960s, vaccination with a formalin-inactivated respiratory syncytial virus (RSV) vaccine candidate in alum increased severity of disease upon subsequent RSV infection [10–14]. Two children died, and postmortem evaluation revealed pulmonary eosinophilia, a hallmark of enhanced respiratory disease (ERD) [10]. Distinct (and non-mutually exclusive) immune phenomena linked to either humoral or cellular responses have been postulated to explain the disease enhancement observed after RSV vaccination [9,15–17]. Suboptimal humoral responses characterized by a high binding to neutralizing antibody ratio have been proposed to favor the deposition of immune complexes, ultimately resulting in increased inflammation [16–19]. Alternatively, cellular responses with a Th2/Th1 balance skewed toward Th2 have been postulated to promote cellular infiltration and subsequently enhanced respiratory disease (ERD) [9,16,17,19].

More recently, a live-attenuated tetravalent dengue vaccine proved to be protective in individuals who were seropositive for dengue prior to vaccination, but increased the risk of hospitalization and severe disease in individuals who were seronegative [20]. This was widely attributed to antibody-dependent enhancement (ADE) of infection, a phenomenon in which sub-neutralizing levels of antibodies increase Fc-receptor-mediated viral entry, resulting in higher viral burden and exacerbated disease. ADE of dengue is not only a concern after vaccination. Epidemiological data have long suggested that ADE is relevant in the context of natural immunity and infection as well [21]. Indeed, ADE is often used to explain why severe dengue disease is more frequent in individuals with a history of exposure to a heterologous dengue serotype, or in infants (around 6–9 months of age) born to dengue-immune mothers [21,22]. Hence, it is important to note that while vaccination against RSV and dengue both exemplified the possibility of vaccine-induced increased pathology upon infection, the mechanisms postulated for disease exacerbation were distinct.

## 2. Coronavirus disease enhancement after experimental vaccination in animals

The possible risk of vaccine-induced disease enhancement for epidemic coronaviruses has been brought to light during pre-clinical studies with SARS-CoV and MERS-CoV vaccine candidates. It is reasonable to suspect that the same could apply to SARS-CoV-2 since SARS-CoV-2 shares a high degree of sequence homology with SARS-CoV and, to some extent, with MERS-CoV [23,24]. Evidence for vaccine-induced disease enhancement upon infection after vaccination with SARS-CoV and MERS-CoV vaccine candidates in animal models is summarized below and in Table 1.

**Table 1.** Summary of SARS-CoV and MERS-CoV vaccine candidates animal studies assessing protection and lung pathology.

Type of vaccine	Virus	Antigen	Adjuvant	Animal	Protection ^(1)^	Lung pathology ^(2)^	Reference
Inactivated	SARS-CoV	Whole virus	-	Mouse (Balb/c, C57BL/6)	Yes	Yes	[25–27]
Inactivated	SARS-CoV	Whole virus	Alum	Mouse (Balb/c, C57BL/6)	Yes	Yes	[25,26,28]
Inactivated	SARS-CoV	Whole virus	Delta inulin	Mouse (Balb/c)	Yes	No	[27]
Inactivated	SARS-CoV	Whole virus	TLR-ligation	Mouse (Balb/c)	Yes	Some	[26]
Inactivated	SARS-CoV	Whole virus	-	Ferret	Some	No	[30]
Inactivated	SARS-CoV	Whole virus	Alum	Ferret	Some	No	[30]
Inactivated	SARS-CoV	Whole virus	-	Hamster	Yes	No	[31]
Inactivated	SARS-CoV	Whole virus	ASO1B	Hamster	Yes	No	[31]
Inactivated	MERS-CoV	Whole virus	-	Mouse (hCD26/DPP4)	Yes	Yes	[29]
Inactivated	MERS-CoV	Whole virus	Alum	Mouse (hCD26/DPP4)	Yes	Yes	[29]
Inactivated	MERS-CoV	Whole virus	MF59	Mouse (hCD26/DPP4)	Yes	Yes	[29]
Virus Like Particle	SARS-CoV	S	-	Mouse (Balb/c)	Yes	Yes	[25]
Virus Like Particle	SARS-CoV	S	Alum	Mouse (Balb/c)	Yes	Yes	[25]
Subunit	SARS-CoV	S	-	Mouse (Balb/c)	Yes	Yes	[25,27]
Subunit	SARS-CoV	S	Alum	Mouse (Balb/c)	Yes	Yes	[25,27]
Subunit	SARS-CoV	S	Delta inulin	Mouse (Balb/c)	Yes	No	[27]
Subunit	SARS-CoV	RBD-Fc	FCA/FIA	Mouse (Balb/c)	Yes	No	[32]
Subunit	SARS-CoV	S trimer	Alum	Hamster	Yes	No	[33]
Adeno vector	SARS-CoV	S + N	-	Ferret	Some	No	[30]
VV or VEE vector	SARS-CoV	S	-	Mouse (Balb/c)	Yes	No	[34,35]
VV or VEE vector	SARS-CoV	N	-	Mouse (Balb/c)	No	Yes	[34,35]
VV vector	SARS-CoV	S	-	NHP	Yes	Yes	[40]
Adeno vector	MERS-CoV	S1	-	Mouse (hDPP4 Tg+)	Yes	Yes ^(3)^	[36]
Adeno vector	MERS-CoV	S1	CD40-L	Mouse (hDPP4 Tg+)	Yes	No	[36]
Live attenuated	SARS-CoV	Whole virus	-	Hamster	Yes	No	[39]
Nucleic acid/protein	MERS-CoV	S cDNA/S1 prot	AlPO4	NHP	Yes	No	[41]
Nucleic acid	MERS-CoV	S DNA	-	NHP	Yes	No	[42]

^(1)^Reduction of lung viral titer and/or increase in survival upon challenge ^(2)^Lung cellular infiltration, mainly eosinophils except in ^(3)^ where lung pathology manifested in the form of perivascular hemorrhage

**Abbreviations**: VV = vaccinia virus, VEE = Venezuelan equine encephalitis replicon, S = spike protein, RBD = receptor-binding-domain, S1 = spike domain 1, N = nucleoprotein, NHP = non-human primate, FCA = Freund’s complete adjuvant, FIA = Freund’s incomplete adjuvant

### 2.1. Inactivated vaccine candidates

In mice, several whole-inactivated SARS-CoV candidates (with and without alum) reduced lung viral titer and/or mortality upon viral challenge, but at the same time induced increased lung immunopathology in the form of eosinophilic infiltration (i.e. unusual presence of eosinophilic cells in the lung tissue) upon infection [25–28]. In some cases, adjuvanting the vaccine with delta inulin or ligation of Toll-like receptors (TLR) successfully limited immune infiltration [26,27]. Interestingly, one study showed that in older mice, protection was lower and eosinophilic immune infiltration was exacerbated as compared to younger mice [28]. Similarly, an inactivated MERS-CoV vaccine in alum or MF59 adjuvant resulted in the production of neutralizing antibody and reduced lung viral titers (upon viral challenge), but induced increased eosinophil infiltration upon homologous coronavirus challenge, despite reducing lung viral titer and/or mortality [29].

In ferrets, vaccination with whole killed SARS-CoV (either alum-adjuvanted or not) induced neutralizing antibodies and reduced viral load, and no disease enhancement was detected upon challenge [30]. In hamsters, vaccination with a whole-virus inactivated SARS-CoV vaccine both adjuvanted with and without AS01B induced neutralizing antibody and protective immunity, and did not induce eosinophil infiltration in the lungs [31].

### 2.2. Viral like particles and subunit

A virus-like particle (VLP) based vaccine composed of SARS-CoV spike (S) protein (that mediates attachment to the receptor on host target cell) and the nucleocapsid (N), envelope (E) and membrane (M) proteins from the murine coronavirus mouse hepatitis virus (MHV) also induced both protection and eosinophil infiltration in the lungs upon challenge in mice [25]. Subunit spike protein vaccine candidates also induced both protection and eosinophilia with and without alum when mice were challenged [25,27], and the immunopathology was reduced when the spike protein was adjuvanted in delta inulin [27]. In contrast, vaccination of mice with SARS-CoV receptor-binding-domain fused to Fc (RBD-Fc) in Freund’s complete adjuvant (FCA) and boosted with RBD-Fc in Freund’s incomplete adjuvant (FIA) induced neutralizing responses and reduced viral load in the lungs upon challenge [32], but no sign of lung pathology was observed. Finally, immunization of hamsters with trimers of the full-length spike protein of SARS-CoV in alum was immunogenic, protective and did not induce lung pathology [33].

### 2.3. Viral vectors and live attenuated

In a murine model, vaccination with viral vectors (such as vaccinia virus or Venezuelan equine encephalitis virus replicons) expressing SARS-CoV spike (S) protein induced protection and no immunopathology upon challenge [34,35]. On the other hand, the reverse (immunopathology and no protection) was observed if the antigen expressed was the nucleocapsid (N) protein [34,35]. Again, protection (but not immunopathology) was reduced in older mice [34]. Furthermore, an adenovirus-based vaccine candidate expressing domain 1 of the MERS spike (S1) protein induced neutralizing antibodies and reduced viral load, but induced lung perivascular hemorrhage upon challenge [36]. The hemorrhage disappeared when the S1 protein was fused with the CD4 T cell co-stimulatory molecule CD40-L [36].

In ferrets models, vaccination with an adenovirus-based vector encoding the S and N proteins of SARS-CoV induced neutralizing antibodies and reduced viral load after challenge, but protection was incomplete. Disease enhancement was not detected upon challenge [30]. In other ferret studies, vaccination with an attenuated modified vaccinia virus Ankara vector expressing either recombinant SARS-CoV S or N proteins not only failed to induce protection from SARS-CoV challenge, but also increased liver pathology upon infection. Lung pathology was not assessed [37,38]. In hamsters, a live-attenuated SARS-CoV strain-induced neutralizing antibody and protective immunity, and did not induce eosinophil infiltration in the lungs [39].

In non-human primates, vaccination with a modified vaccinia virus encoding the full-length SARS-CoV S protein induced high levels of neutralizing antibodies and reduced viral load in oral swabs upon infection [40]. Viral challenge of vaccinated animals also induced lung pathology in the form of eosinophil infiltration compared to mock-vaccinated animals. Furthermore, this study showed that the transfer of vaccine-induced IgG prior to infection increased lung pathology and cell infiltration upon infection, linking vaccine-induced antibodies to increased lung pathology *in vivo*.

### 2.4. Nucleic acid vaccines (RNA and DNA vaccines)

Immunization of NHPs with full-length MERS-CoV S protein cDNA (twice), followed by a boost with S1 protein in AlPO4 resulted in the production of neutralizing antibodies, and protected animals from severe lung infiltrate upon infection [41]. Similar results were obtained after vaccination of NHPs with a synthetic DNA vaccine encoding the MERS-CoV S protein [42]. Nucleic acid vaccines are particularly attractive in epidemic situations, among others due to their relatively rapid manufacturing process [3]. Several RNA and DNA vaccines against SARS-CoV-2 are under development and three have entered human clinical testing [5]. Current preclinical testing of these platforms in animal models should provide more knowledge in the upcoming months.

## 3. Knowledge gaps

While data in some animal models with different SARS-CoV and, to a lesser extent, MERS-CoV vaccines have provided evidence of enhanced pulmonary disease, the studies have not necessarily presented a consistent picture. In addition, the mechanisms triggering enhanced pathology remain elusive. Involvement of non-neutralizing antibodies, Th2-skewing of the Th2/Th1 balance (either increased Th2 or decreased Th1) and sub-optimal CD8 responses have all been postulated to explain vaccine-induced disease enhancement [26,27,40,43,44]. Importantly, the biological differences between SARS-CoV and SARS-CoV-2 require that comparable animal studies be performed with SARS-CoV-2 vaccine candidates before conclusions can be drawn. Similarly, the natural history of COVID-19 in humans and epidemiological determinants of severe disease needs to be explored to guide the vaccine development process.

### 3.1. CoV vaccine design and vaccine-induced immune responses

A more systematic understanding of the impact of the vaccine-type, antigen choice, and adjuvant selection on protection (or enhancement) would be helpful to rationalize vaccine development efforts. Due to the heterogeneity of the experiments performed (different vaccine types, adjuvants, and antigens) and the variety of animal models used (some of which might be inherently prone to more or less severe coronavirus-induced pathology), drawing clear conclusions is currently difficult. However, a few interesting points are worth mentioning. For example, lung pathology in the form of eosinophilic infiltration was frequently observed with whole-inactivated vaccines in alum in mice. Alum is widely thought to promote Th2 immune responses in mice, but in humans, it might simply be a poor inducer of cellular immune responses [45]. Therefore, these observations in mice may not be mirrored in humans. On the other hand, lung pathology was not observed when delta inulin was used as an adjuvant with whole inactivated or subunit vaccine candidates in mice [27]. Interestingly, delta inulin is known to promote robust cellular responses, and a balanced Th1/Th2 response [46]. Similarly, TLR-agonists were able to prevent Th2-skewing (and lung pathology) when given with UV-inactivated whole virions [26].

With respect to the antigen choice, head-to-head comparisons of vaccines expressing either spike or nucleoprotein suggest that nucleoprotein offers less protection and causes more lung pathology in mice [34,35], but additional data in other species and/or with other vaccine platforms will be necessary to better inform antigen choice. Similarly, a better understanding of the immunogenicity and protective effect of different domains of the spike (or other) viral proteins is needed. Systematically comparing vaccine types, adjuvants, and antigens in the same animal model might shed some light on the potential skewing of the immune response toward protection or pathogenesis.

In animals, several SARS-CoV and MERS-CoV vaccine candidates induced a neutralizing response, mediated protection, yet increased lung infiltration and/or pathology at the same time (see Table 1). Currently, the first preclinical animal studies on vaccination with SARS-CoV-2 candidates are being published. A recent study in non-human primates demonstrated the protective efficacy of several prototype DNA vaccines expressing variants of the SARS-CoV-2 S protein [47]. Enhanced clinical disease was not observed after vaccination, but the authors cautioned that the study was not designed to address safety issues, and advise that further studies are required to investigate the question of possible enhanced respiratory disease. Another study in non-human primates vaccinated with inactivated SARS-CoV-2 adjuvanted with alum showed no evidence of enhanced pulmonary disease after challenge [48]. While these NHP studies are reassuring, it is currently unknown whether these observations will also apply to humans. Disentangling protection and lung pathology after vaccination will be necessary to develop a safe and efficacious vaccine against SARS-CoV-2, in particular for individuals who are already naturally at higher risk of severe COVID-19 disease, such as older population or in the presence of co-morbidities [49,50].

Finally, the potential cross-protection that each SARS-CoV-2 vaccine candidate could confer against SARS-CoV and, perhaps more importantly, against potential future emerging SARS-CoV-X remains a crucial question. Answering it would channel development efforts toward vaccine candidates that not only safely protect against SARS-CoV-2, but also have the potential to mitigate putative future epidemics of as-yet-unknown SARS-CoV-X.

### 3.2. Pathogenesis and immunity in humans

There is currently no evidence in humans that preexisting immunity to another (or the same) coronavirus can increase the severity of COVID-19 disease. A recent study has shown that human polyclonal antibody induced by SARS-CoV infection could bind to SARS-CoV-2, and vice versa [51]. No (or very little) cross-neutralization was observed. However, another study reported neutralization of SARS-CoV-2 pseudo-virus by serum from convalescent SARS-CoV patients [52]. Additional studies will be required to further investigate cross-reactivity, cross-neutralization and/or cross-protection between SARS-CoV and SARS-CoV-2, and in particular to clarify whether preexisting cross-reactive antibodies have a protective or detrimental effect on subsequent infections.

With respect to disease severity during human infection, it is interesting to note that seasonal (low pathogenic) coronaviruses such as HCoV-229E, HCoV-OC43, HCoV-NL63, and HCoV-HKU cause mild self-resolving cold-like disease. In contrast, epidemic (highly pathogenic) coronaviruses such as MERS-CoV, SARS-CoV and more recently SARS-CoV-2 can cause severe pneumonia, acute lung injury (ALI), or even acute respiratory distress syndrome (ARDS), resulting in high morbidity and mortality [53,54]. The exact mechanisms that drive protection versus severe pneumonia are not yet fully understood. However, protective immunity and recovery after mild infection is typically characterized by ‘regulated’ inflammation, early IFN-γ production, limited virus replication, and optimal B and T cell responses. In contrast, severe outcomes are associated with rapid viral replication, delayed IFN-γ responses, lymphocyte infiltration of the lungs, excessive pro-inflammatory cytokine production, suboptimal T cell responses (including Th2-skewing) which act in concert to induce tissue damage, severe respiratory outcomes, sometimes leading to death [53–55]. After SARS-CoV-2 infection, increased levels of cytokines such as IL-6, IL-10 and TNFα, low CD4 and CD8 T cell counts, and decreased IFN-γ expression in CD4 T cells were hallmarks of severe COVID-19 disease [49,56,57]. Recently, unusually high numbers of children with multisystem inflammatory syndrome have been reported, and a possible link to SARS-CoV-2 infection has been postulated [58,59].

It is necessary to better understand the pathogenesis and immunity induced by natural infection. Serological profiles, cellular signatures, cytokine combinations, and/or gene expression patterns that correlate with the severity of disease should be defined. For example, the relationship between antibody and disease outcome should be investigated, to clarify the contribution of antibody isotypes, sub-classes, effector functions, and/or epitope-specificity to protection or exacerbation of pathology. Also, considering SARS-CoV-2 is a respiratory virus, surprisingly little is known about the presence and potential role of IgA after infection. The relative contribution of humoral and cellular immunity to disease severity will also need to be elucidated. In particular, a better grasp of how Th1/Th2 skewing impacts infection outcome is needed. Defining markers, correlates, and/or predictors of disease severity and/or outcome in natural infection that can be extrapolated to vaccination is crucial. If these are valid both in animals and in humans, they would be of paramount importance to down-select vaccine candidates, and advance safer ones along the clinical development process.

### 3.3. Epidemiological considerations

Epidemiological evidence of the association between host factors and disease outcome (or vaccination outcome) should also be taken into consideration. Age, gender, and comorbidities are all potential determinants of disease outcome (and presumably lung pathology) after natural infection, but their relative contribution is still a matter of debate [50]. It is reasonable to speculate that these same factors (and others such as genetic makeup and pre-vaccination immune status) will influence immune responses after vaccination. Who should the vaccine be given to, and what host factors are susceptible to increase or decrease vaccine-mediated protection or putative disease enhancement? These crucial questions have been touched upon, but remain partly unanswered.

Another important question is the degree of cross-reactivity and cross-protection for humoral and cellular responses within epidemic coronaviruses (such as SARS-CoV, SARS-CoV-2, MERS-CoV) and with seasonal hCoV. Will a vaccine for SARS-CoV-2 protect against future emerging SARS-CoV-X? Similarly, the impact of preexisting immunity to SARS-CoV, MERS-CoV, or seasonal hCoV on SARS-CoV-2 infection or vaccination remains elusive. The possible modulation of vaccine responses (both protective and potentially enhancing) by the sero-status for seasonal and/or epidemic coronaviruses at the time of vaccination is unclear. Understanding the impact, if any, of prior human coronavirus exposure on vaccination outcomes may be an important consideration that should be explored. Sero-epidemiology studies could be performed to understand the prevalence of antibodies against seasonal and/or epidemic coronaviruses (and the potential cross-reactivity between the two) in different age-groups and risk populations.

## 4. Conclusion

As the world is rushing for a COVID-19 vaccine, the possibility of vaccine-mediated disease enhancement has been flagged as a potential safety concern. Here, we have summarized available animal data on immunogenicity, protection, and lung pathology of vaccine candidates for SARS-CoV and MERS-CoV, which are both related to SARS-CoV-2. We have also highlighted knowledge gaps and suggested research areas worth exploring to inform and de-risk the development of a vaccine against COVID-19.

## 5. Expert opinion – the way forward

In the light of the current COVID-19 pandemic and the high social and economic cost of mitigation plans, there is a sense of urgency in developing efficacious countermeasures. While rapidly building a solid diagnostic, therapeutic, and vaccine arsenal is an absolute priority, safety should not be shrouded by the extraordinary nature of the situation [60]. This is particularly true for vaccines, and even more so for a coronavirus vaccine, where the possibility of vaccine-mediated disease enhancement has been voiced [6–8].

The mechanisms leading to vaccine-induced disease enhancement (if enhancement happens in humans at all) for coronaviruses may be partly (or entirely) different from the mechanisms which resulted in increased pathology after RSV vaccination, which in turn were largely distinct from the ones postulated for dengue. While lessons learned from RSV and dengue will be extremely helpful in developing a safe vaccine against SARS-CoV-2 (and future SARS-CoVs), there is no ‘one size fits all’ solution to the question of potential vaccine-mediated disease enhancement. SARS-CoV-2-specific information, tools, and approaches are required to adequately fill existing knowledge gaps. A more comprehensive understanding of the host and viral determinants of protection versus pathology during SARS-CoV-2 infection (or after vaccination) would enable a less empirical and safer vaccine development process.

Numerous animal models exist for SARS-CoV and MERS-Cov infections [61,62]. Animal models may be important in understanding and de-risking disease enhancement [7,8]. Understanding and refining them, using the appropriate models to answer the question of putative enhancement, and ensuring that pre-clinical findings can be extrapolated to humans would maximize the usefulness of animal models in mitigating the risk of vaccine-induced disease enhancement for SARS-CoV-2. Ideally, the risk of disease enhancement should be investigated in multiple animal models. Comparing different well-characterized animal models is important because some species or strains may be inherently prone to more or less severe CoV pathology. Therefore, depending on the experimental system used, the risk of vaccine-mediated enhancement might be magnified or masked.

Prior to clinical development, the potential for vaccine-induced disease enhancement should be ‘evaluated according to current science available, which may include the use of relevant animals model currently in development’ [8]. However, with the current surge in COVID-19 related research, experimental animals might be in short supply [63], and delaying first-in-humans (FIH) trials until animal experiments are performed might not be universally considered a practical option. Recently, a group of regulatory experts convened by the ICMRA generally agreed (but not unanimously) that for some vaccine candidates for which there is extensive knowledge around the immune response elicited, FIH trials (phase I) may be allowed to proceed in the absence of animal experiments addressing potential disease enhancement, as long as adequate risk mitigation measures exist for these FIH trials [8]. However, animal experiments addressing the issue should be performed alongside the FIH trials, and in any case, done before larger phase II and III trials.

Ultimately, even the absence of vaccine-mediated disease enhancement in animals might not entirely exclude the possibility of enhanced disease in humans. Therefore, from early clinical stages onwards, assessment of disease enhancement should be an explicit objective in trials, as suggested by the WHO [6]. Clinical trials should be deliberately designed to detect safety signals at early stage and include an adequate long-term follow-up. This is particularly important to detect whether waning immune responses may allow the unmasking of clinically relevant disease enhancement phenomenon. Finally, should COVID-19 cases be detected during trials, the biomarkers, clinical signs, and symptoms associated with severe disease should be thoroughly documented [49,57]? Also, as preexisting antibodies have been suggested to be involved in the disease enhancement process (at least for dengue and possibly for RSV), a pre-vaccination serum sample should imperatively be collected in all subjects to be able to analyze pre-vaccination sero-status if necessary.

The urgency engendered by the pandemic has resulted in acceleration of the clinical vaccine development process from 5–10 years to 12–18 months. In part, there will be telescoping of sequential processes in the human Phase I to III clinical trials, but data-driven efficacy outcomes will likely be similar to what is seen in the normal process. On the human safety side, however, collection of safety outcomes, particularly longer-term outcomes related to SARS-CoV-2 exposure, may be less complete at dossier submission than is typically present under normal circumstances. However, as long as the standard use of data and safety monitoring boards prevails, longer-term disease surveillance can be incorporated into study design (and into Phase IV effectiveness trial designs). Safety data collected in the shorter term and subsequently accumulated over time will mirror what is collected in traditional vaccine clinical development. The continuous, systematic, and actively monitored collection of safety data in vaccinated humans should allow for prompt and appropriate detection of safety signals both during the Phase I to III clinical trials and after completion of efficacy trials.

The global response to COVID-19 has generated an unprecedented amount of knowledge, at an unprecedented speed. As a result, new diagnostic tests, therapeutics, and vaccines have moved along the development pipeline in just a few months. This has in part been possible thanks to widespread and rapid data sharing either directly, via national and international stakeholders, or through online data and/or manuscript repositories. Data sharing has been hailed as a pillar of public health action [64], and will hopefully continue throughout the current and future crises. With respect to the risk of disease enhancement, rapid data sharing could act as an ‘early warning system’ to alert the scientific community should a safety signal arise in a trial. For example CEPI, which funds multiple vaccine candidates for SARS-CoV-2, has partnered with the Brighton Collaboration, an organization that facilitates the development, evaluation, and dissemination of information about vaccine safety. Together, CEPI and the Brighton Collaboration launched the Safety Platform for Emergency Vaccines (SPEAC) to help manufacturers oversee the safety profile of their vaccine candidates.

In the current COVID-19 pandemic context, the potential benefits of a safe and efficacious vaccine are evident. A vaccine would be instrumental to protect at-risk populations, curb the epidemic and, should it be protective against not-yet-known coronaviruses, dramatically improve preparedness for future outbreaks, reducing their human and economic footprint. However, if a coronavirus vaccine was to exacerbate disease upon subsequent infection, it would have disastrous consequences, as recently exemplified by the public anxiety caused by a dengue vaccine increasing the risk of severe disease in seronegative individuals [65,66]. The presence (or even the suspicion) of vaccine-mediated disease enhancement (if it were to happen) would erode confidence in a coronavirus vaccine, and perhaps vaccines in general. Vaccine confidence is necessary to guarantee high uptake and maintain adequate coverage required to preserve herd immunity and/or curb epidemics. This is particularly true in high-risk situations, and a break in vaccine trust before or during a pandemic would be regrettable [65]. Both infectious diseases of epidemic potential (such as a global influenza pandemic, Ebola or other high-threat pathogens) and vaccine hesitancy have been flagged by the WHO as major threats to global health in 2019 [67].

In the midst of the current COVID-19 pandemic, developing a vaccine as soon as possible is a clear imperative. But it is also a balancing act between what we know and what we do not know, and efforts should focus on generating necessary information without imposing unacceptable delays on the development process. Despite the unprecedented global humanitarian crisis, safety and scientific rigor cannot not be sacrificed to speed. We must be mindful of the risk raised by animal studies of other SARS-CoV and MERS-CoV vaccines and apply these learnings to the development of a safe and effective vaccine against SARS-CoV-2.

## Article highlights

- As governments around the world have taken drastic public health measures to limit the spread of SARS-CoV-2 and minimize morbidity and death due to COVID-19 disease, there is an urgent need for a safe and efficacious SARS-CoV-2 vaccine.
- The theoretical risk of vaccine-mediated disease enhancement with coronavirus vaccines has been flagged by the scientific community as a potential safety concern and should be thoroughly assessed despite the urgency of the situation.
- This article reviews the evidence suggesting a theoretical risk of disease enhancement after vaccination with SARS-CoV and MERS-CoV vaccine candidates in animal models. Subsequently, we delineate knowledge gaps that need to be addressed to inform the vaccine development process and minimize the risk of encountering vaccine-mediated disease enhancement with upcoming SARS-CoV-2 candidates.
- We propose studies to fill these knowledge gaps and present avenues to investigate the risk of vaccine-mediated disease enhancement during both pre-clinical and early clinical development stages.
- Despite the urgent need for a vaccine, safety should remain of critical importance during the development of a SARS-CoV-2 vaccine.
